# Supplementary material for: Universalities of thermodynamic signatures in topological phases
Source: Sci Rep. 2016 Dec 8;6:38530. doi: 10.1038/srep38530 (PMC5144081; doi:10.1038/srep38530)
Supplement: Supplementary Information [file srep38530-s1.pdf]

Universalities of thermodynamic signatures in topological phases
Supplementary information

S.N. Kempkes[1], A. Quelle[1], and Cristiane Morais Smith[1,*]

[1]Institute for Theoretical Physics, Center for Extreme Matter and Emergent Phenomena, Utrecht University, Princetonplein 5, 3584 CC Utrecht, The Netherlands
[*]c.demoraissmith@uu.nl

September 4, 2016

Results SSH model

The SSH model is a 1D model, which was first used to describe the dimerization of polyacetylene[1]. It describes electrons hopping between two neighboring sites of a bipartite lattice, and has proven to be an adequate tight-binding model for capturing the behavior of conjugated polymer chains. The SSH Hamiltonian reads

$$H_{\mathrm{SSH}} = \sum_n \left[t_1 c^\dagger_{A,n} c_{B,n} + t_2 c^\dagger_{A,n+1} c_{B,n} + h.c. \right], \tag{1}$$

where t_1 and t_2 denote unequal hopping amplitudes between the even and odd links for the even (odd) sites A (B). It is common to rewrite $t_1 = t(1+\Delta)$ and $t_2 = t(1-\Delta)$, which makes the Hamiltonian only dependent on the parameter Δ, and thus there is a phase change between the topological and trivial phases for $\Delta = 0$. In Fig. 1, we show the results for the SSH chain for $t = 3/2$. We observe a first-order phase transition at the edge and a second-order phase transition in the bulk, which is precisely the same behavior obtained for the Kitaev chain.

Upon increasing the temperature, the phase transition smooths out. In Fig. 2 we compare the finite-T phase diagram with the ones obtained via the Uhlmann phase[2], using the same procedure as for the Kitaev chain. Again, the comparison between the two results is clear.

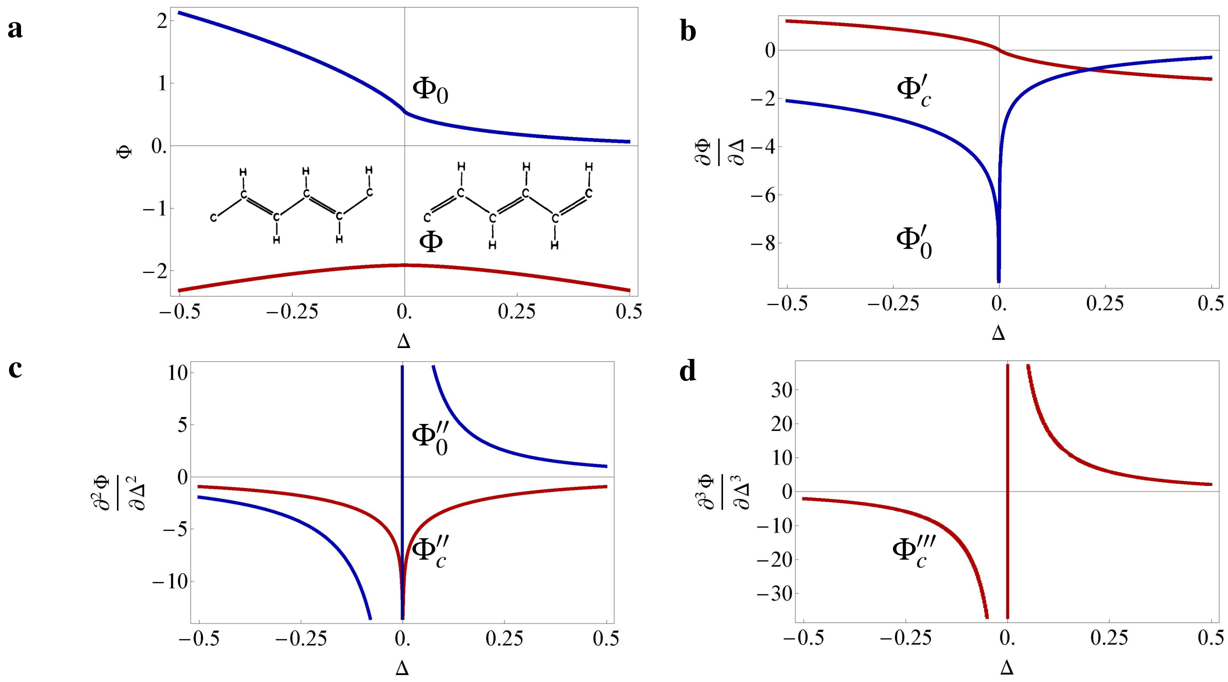

Figure 1. Behavior of the thermodynamic potentials and their derivatives for the SSH model. a The conventional potential Φ_c (red) and subdivision potential Φ_0 (blue) and (**b, c, d**) their derivatives with respect to Δ, for with $t = 3/2$. The figures show a similar behavior as the Kitaev chain, a first-order phase transition for the edge and a second-order phase transition in the bulk. The inset in **a** shows the topological (left) and trivial (right) phase of polyacetylene.

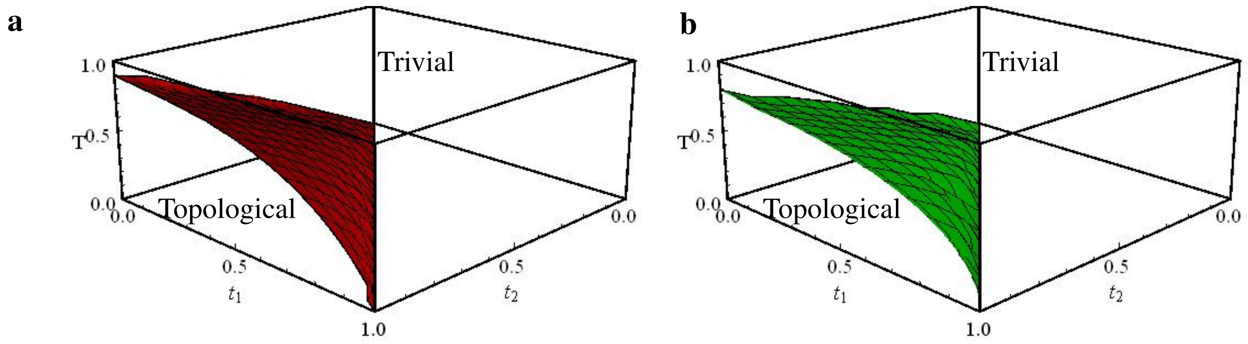

Figure 2. Finite-T phase diagram SSH model The finite-T phase diagram for the SSH model obtained via **a** Hill thermodynamics (red) and **b** via the Uhlmann phase (green)[2].

References

1. Su, W. P., Schrieffer, J. R. & Heeger, A. J. Solitons in polyacetylene. *Phys. Rev. Lett.* **42**, 1698–1701 (1979).
2. Viyuela, O., Rivas A. & Martin-Delgado, M. A., Uhlmann Phase as a Topological Measure for One-Dimensional Fermion Systems. *Physical Review Letters* **112**, 130401(2014).
